# Autophagy Modulation by Viral Infections Influences Tumor Development

**DOI:** 10.3389/fonc.2021.743780

**Published:** 2021-10-22

**Authors:** Lucas Leonardi, Sophie Sibéril, Marco Alifano, Isabelle Cremer, Pierre-Emmanuel Joubert

**Affiliations:** ^1^ Institut National de la Santé et de la Recherche Médicale (INSERM), UMRS1138, Centre de Recherche des Cordeliers, Paris, France; ^2^ Sorbonne Université, Univ Paris, Paris, France; ^3^ Department of Thoracic Surgery, Hospital Cochin Assistance Publique Hopitaux de Paris, Paris, France

**Keywords:** tumor development and progression, autophagy, viral infection, oncolytic virus, oncogenic virus, immunity, tumor resistance, tumorigenesis

## Abstract

Autophagy is a self-degradative process important for balancing cellular homeostasis at critical times in development and/or in response to nutrient stress. This is particularly relevant in tumor model in which autophagy has been demonstrated to have an important impact on tumor behavior. In one hand, autophagy limits tumor transformation of precancerous cells in early stage, and in the other hand, it favors the survival, proliferation, metastasis, and resistance to antitumor therapies in more advanced tumors. This catabolic machinery can be induced by an important variety of extra- and intracellular stimuli. For instance, viral infection has often been associated to autophagic modulation, and the role of autophagy in virus replication differs according to the virus studied. In the context of tumor development, virus-modulated autophagy can have an important impact on tumor cells’ fate. Extensive analyses have shed light on the molecular and/or functional complex mechanisms by which virus-modulated autophagy influences precancerous or tumor cell development. This review includes an overview of discoveries describing the repercussions of an autophagy perturbation during viral infections on tumor behavior.

## Autophagy

**Figure 1:**
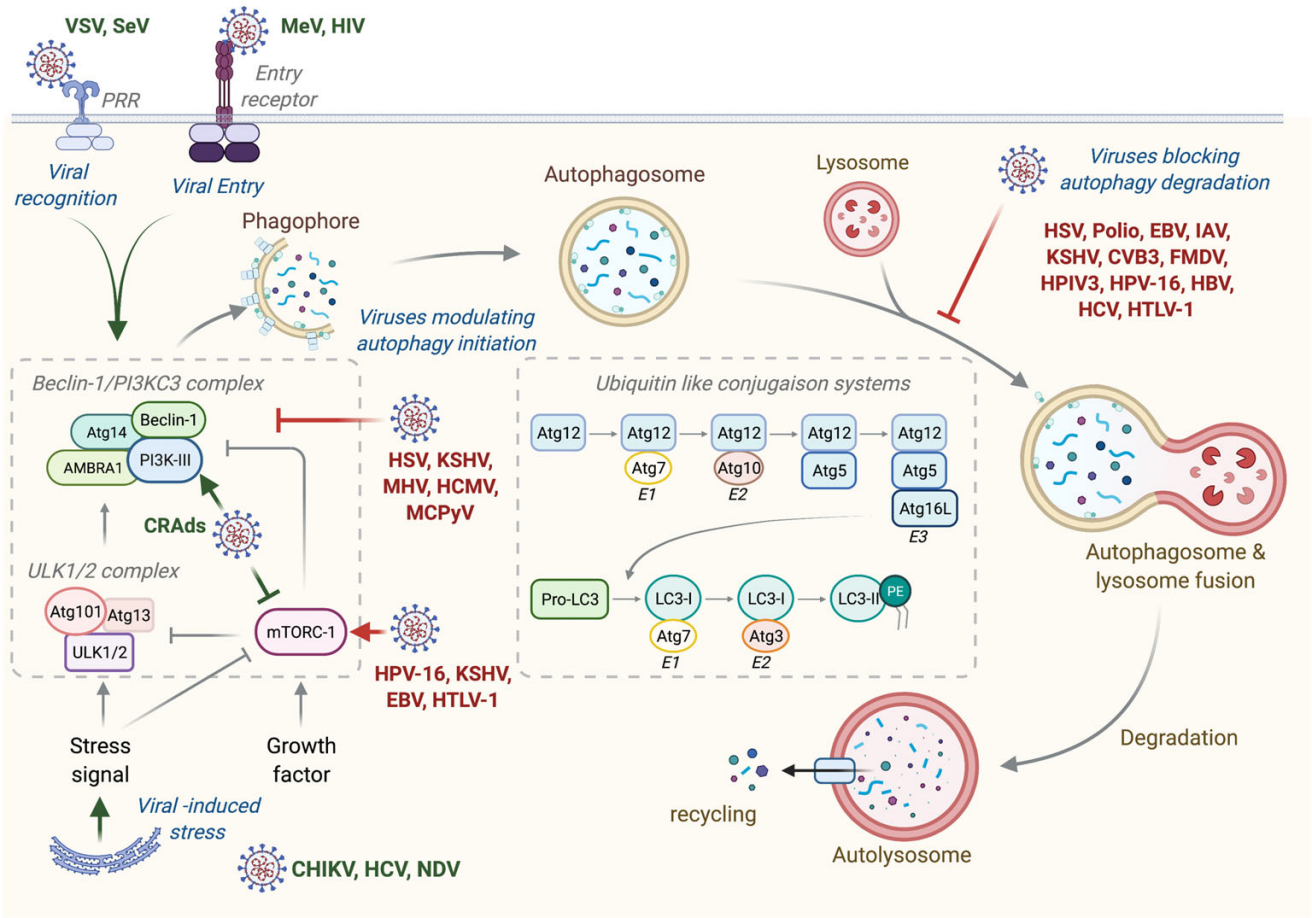
Autophagy machinery and its modulation by viruses. Beclin-1/PI3KC3 complex activation, which is regulated by different mechanisms including the ULK1/2 complex and mTORC1 complex, results in the induction of an autophagic vesicle, which is characterized by a double-membrane, named autophagosome. Two ubiquitin-like systems are essential for autophagosome formation. In the first, autophagy-related gene-12 (Atg12) is conjugated to Atg5, together forming a complex with Atg16L1, which decorates the outer membrane of the phagophore. Microtubule-associated protein 1 light chain-3 (LC3, also known as Atg8) constitutes the second ubiquitin-like system and conjugates phosphatidylethanolamine (PE) at the outer and inner autophagosomal membrane. Unlike the Atg12/Atg5/Atg16L1 complex that is recycled by the protease Atg4, the LC3-PE (referred to LC3-II) remains associated with the inner membrane of autophagosome. The incorporation of phospholipid into the autophagosome membrane is essential for its elongation and regulates the membrane transport system. Autophagosome maturation is characterized by the formation of an autolysosome, the product of fusion with the lysosome. Viruses activate or inhibit autophagy at several step, as indicated on the figure. VSV, Vesical Stomatitis Virus; SeV, Sendai Virus; MeV, Measles Virus; HIV, Human Immunodeficiency Virus; CRads, Conditionally Replicating Adenoviruses; HSV, Herpes Simplex Virus; KSHV, Kaposi’s Sarcoma-associated Herpesviru*s*; MHV, Mouse Hepatitis Virus; HCMV, Human Cytomegalovirus; MCPyV, Merkel Cell Polyomavirus; HPV-16, Human Papillomavirus 16; EBV, Epstein-Barr Virus; HTLV-1, Human T cell Leukemia/lymphoma Virus type 1; CHIKV, Chikungunya Virus; HCV, Hepatitis C Virus; NDV, Newcastle Disease Virus; Polio, Poliovirus; IAV, Influenza A Virus; CVB3, Coxsackievirus B3; FMDV, Foot and Mouth Disease Virus; HPIV3, Human Parainfluenza Virus 3; HBV, Hepatitis B Virus.

Autophagy is a general term defining a catabolic mechanism present in all eukaryotic cells that leads to three different intracellular routes to the degradation of substrates present in the cell by the lysosome. It includes chaperone-mediated autophagy (CMA), microautophagy, and macroautophagy. CMA is the way by which cells degrade intracellular proteins by their translocation to the lysosome through the interaction of chaperone recognizing universal motif in the sequence of CMA substrate (KFERQ motif) and the lysosome protein LAMP-2A (1). Microautophagy involves a direct engulfment of cargos through the invagination of the lysosome membrane or through the direct entry of cytoplasmic materials to the multivesicular bodies of late endosomes (1). Macroautophagy, referred hereafter as autophagy, is a multistep process consisting of the formation of phagophore that elongates, engulfs targeted proteins or organelles in a double-membrane vesicle called autophagosome, and finally fuses with lysosome (**Figure 1**) (2). This process is orchestrated by more than 30 autophagic proteins (Atg), organized in complex. Autophagic induction is modulated by two protein complexes: the ULK1/2 (unc51-like autophagy activating kinase) and the Beclin-1/PI3KC3 (class III phosphatidylinositol 3-kinase) complexes. Once activated, these complexes recruit other proteins involved in elongation and formation of autophagosomes, including the two conjugated systems comprising Atg12-Atg5-Atg16L and LC3. After completion, the mature autophagosome fuses with lysosome to form autolysosome, wherein the sequestered materials and organelles are degraded by lysosomal enzymes (**Figure 1**). Even if autophagy has often been considered as a non-selective mechanism, many studies highlighted an important role for autophagy in selective materials and/or organelles recycling, including mitophagy, which selectively targets damaged mitochondria to autophagosome, or xenophagy, which permits the selective degradation of pathogens and/or pathogens’ elements through autophagy (2).

**Figure 2:**
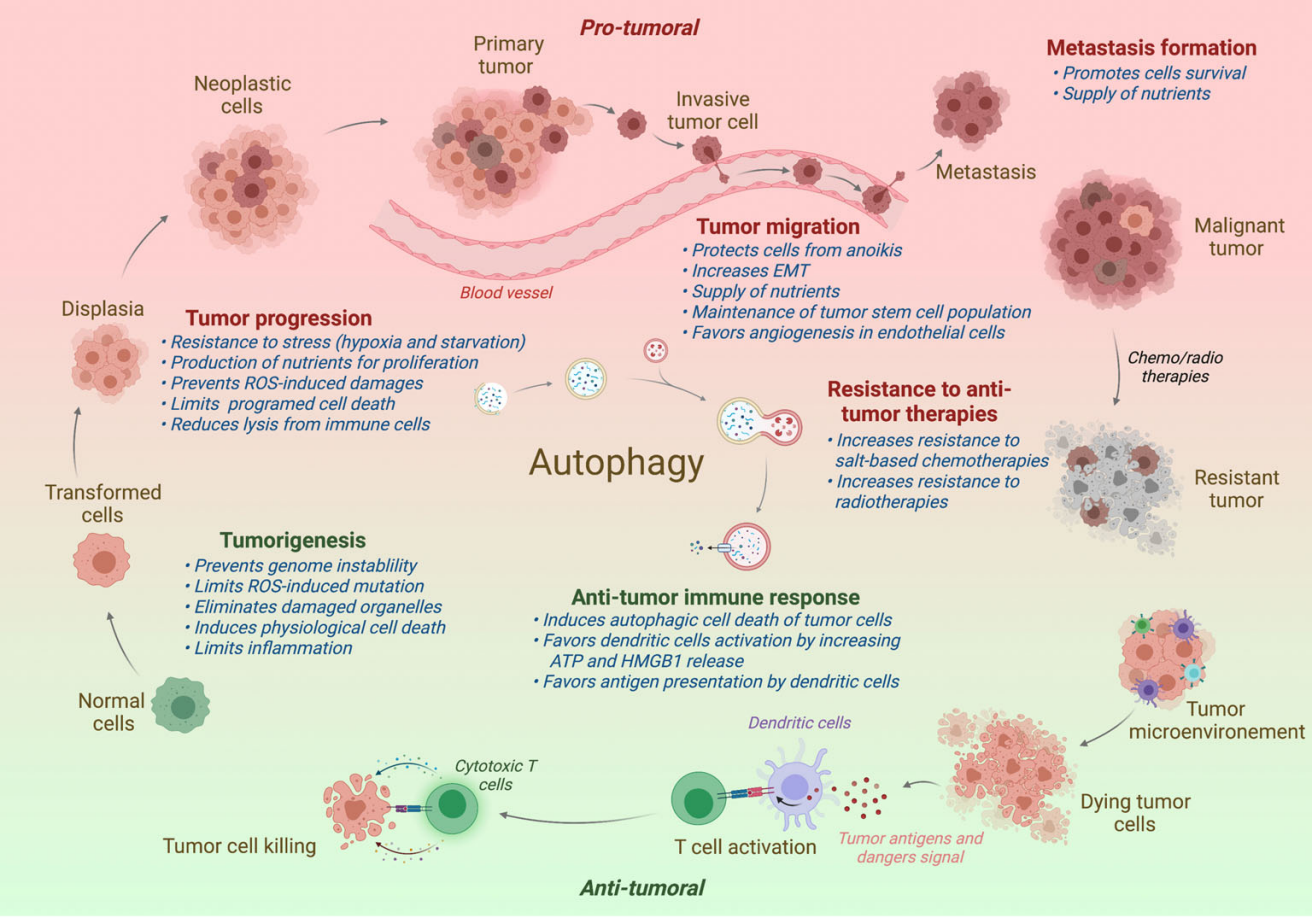
Impact of autophagy on tumor progression. In normal cells, autophagy restricts the tumorigenesis by limiting genome instability and strong inflammation. However, when the tumor is established, autophagy has been shown to promote tumor development, including the tumor progression, migration, and the formation of metastasis. Autophagy has also been associated to a better resistance to antitumor radio- or chemotherapies. In contrast to support tumor, autophagy favors the antitumor immune responses, promoting the immunogenic cell death and the presentation of tumor antigens to the adaptive immune cells. Protumor effects of autophagy are written in red and antitumor effects in green. Effect of autophagy (blue) is indicated at different steps of tumor development. ROS, Radical oxygen species; EMT, Epithelial-Mesenchymal Transition.

Since its discovery by Christian Deduve in 1963, our knowledges on autophagy and its role in physiology has greatly been increased. Autophagy acts as a quality control mechanism by degrading and recycling damaged or old proteins and organelles. Given its important role in homeostasis, it is not surprising that a defect of autophagy has been associated with various pathologies, including neurodegenerative diseases, infection susceptibility, aging, metabolic disorders, and cancer. In cancer, autophagy seems acting as a double-edged sword: in one hand, autophagy limits the tumorigenesis of precancerous cells, and in another hand, it serves as an important survival mechanism for established tumors. The role of autophagy in cancer will be detailed in the different parts of this review and is illustrated in **Figure 2**.

## Autophagy and Viruses

As an intracellular parasite, a virus’s behavior is closely linked to its capacity to prevent and/or subvert cellular antiviral responses. Given that viral infection and replication cause cellular stresses, autophagy is frequently associated with a by-product of infection. However, viral infection-induced autophagy is not a passive process, and an increasing number of studies have shown that autophagy has both antiviral and proviral capacities for the replication of a broad range of viruses (3).

### Viral Infections Modulate Autophagy

Any single step of viral life cycle, including virus binding, entry and recognition, membrane fusion, exposure of viral component, and replication or even antiviral responses, may modulate autophagy (**Figure 1**) (4–6).

The first evidence demonstrating that virions binding could induce autophagy has been observed following measles virus (MeV) infection (7). CD46, a complement family receptor, serves as a binding and entry receptor for vaccinal or attenuated strains of MeV (8, 9). The team of Mathias Faure demonstrated that MeV/CD46 binding leads to an induction of autophagy through a direct interaction of CD46 with the Beclin-1/PI3KC3 using a scaffold protein named Golgi associated PDZ and coiled coil motif-containing (GOPC) (7). Further investigations showed that MeV also activates autophagy by targeting an autophagy-associated protein called immunity-associated GTPase family M (IRGM) (10, 11). Human immunodeficiency virus type 1 (HIV-1) can also activate autophagy in uninfected CD4 T cells through the fusiogenic activity of envelope glycoproteins gp120 and gp41 (called Env) (12). While specific pathway has not been really elucidated, it is important to note that the signaling activity of CD4 and CXCR4 are not associated with autophagy induction in infected cells, in contrast to the fusiogenic activity of Env proteins (12).

In addition to binding and membrane fusion, a broad range of studies have demonstrated an induction of autophagy following the virus recognition. The delivery of viral elements into the cytosol can lead to the induction of autophagy through the activation of pattern recognition receptor (PRR). Among PRR, intracellular endosomal Toll-like receptors (TLRs) are important sensors of viral components, including single-stranded (ss)RNA (e.g., TLR7 and TLR8), double-stranded (ds)RNA (e.g., TLR3), and DNA with CpG site (e.g., TLR9). The majority of TLRs recruit the adaptor myeloid differentiation primary response protein MyD88, whereas TLR3 and TLR4 recruit the TIR domain-containing adaptor molecule 1 (TRIF). In all cases, TLR stimulation leads to the activation of nuclear factor-kB (NF-kB) for the production of inflammatory cytokines and interferon (IFN) production (13). TLR stimulation also activates autophagy, presumably by promoting the binding of both MyD88 or TRIF to Beclin-1 (14). The role of TLR-induced autophagy in virus remains to be determined, but several studies have shown an important impact of this process on antiviral immune responses (4). For instance, plasmacytoid dendritic cells (pDCs) deficient for Atg5 exhibited a reduced TLR-dependent production of IFNs after infection with vesicular stomatitis virus (VSV), sendai virus (SeV), or herpes virus type 1 (HSV-1) (15). The RIG-I like receptors (RLRs) retinoic acid-inducible gene I (RIG-I), which senses dsRNA in the cytosol, also induces autophagy following sendai virus infection (16). RIG-I-mediated autophagy is regulated by the translocation of beclin-1 into the mitochondria dependently of the mitochondrial antiviral signaling protein (MAVS) and tumor necrosis factor receptor-associated factor (TRAF) 6 (16). Protein Kinase R (PKR), which recognizes dsRNA, also triggers autophagy by the phosphorylation of the eukaryotic translation initiation factor (EiF) 2 (17). During HSV-1 infection, the cytosolic DNA sensor cyclic GMP_AMP (cGAMP) synthase (cGAS), which recognizes dsDNA, triggers autophagy through the activation of the initiation complex, releasing Rubicon (an autophagy inhibitor) from the beclin-1/PI3KC3 complex (18, 19).

Viral replication causes drastic modifications to cell homeostasis, which often enhance autophagy. As a good example, chikungunya virus (CHIKV) replication induces autophagy through the synergy of both endoplasmic reticulum (ER) and oxidative stresses (8). CHIKV proteins accumulation promotes ER stress and unfold protein responses (UPR) *via* the activation of inositol-requiring Ser/Thr protein kinase/endonuclease (IRE) 1a. CHIKV also increases the production of reactive oxygen species (ROS) and reactive nitrogen species (NOS), which stimulates autophagy by activating AMP-activated protein kinase (AMPK)-mediated inhibition of mammalian target of rapamycin complex 1 (mTORC1) (20). Similar observation has been reported during HCV infection, whose replication enhances ER and UPR responses that lead to autophagy induction (21).

### Autophagy During Viral Infection: A Double-Edged Sword

Due to its role in cytosolic materials clearance, autophagy can degrade viral components, including viral particles or proteins or even host factors required for the replication of the virus. Indeed, autophagy is considered as an important part of innate antiviral responses, especially in non-immune cells. However, this virus-specific autophagic degradation, also known as virophagy, can be subverted by many viruses.

The first example of virophagy has been observed during Sindbis virus (SINV) infection, in which Beclin-1 and Atg5 protect against viral infection and finally against SINV-mediated encephalitis (22). Mechanistically, SAMD-specific E3 ubiquitin protein ligase 1 (SMUF1) and Fanconi anemia group C protein (FANCC) are both required for the delivery of SINV capsid protein into the autophagosome, through the interaction of the autophagy receptor p62 with SINV proteins (23, 24). SMURF1 and FANCC are also involved in the targeting of herpes simplex virus -1 (HSV-1) proteins for autophagic degradation (24). During poliovirus infection, galectin 8 restricts viral infection through the initiation of autophagic degradation of the viral RNA genome (25). Autophagic degradation of the viral non-structural protein A5 (NS5A) of hepatitis C virus (HCV) has been observed in infected cell through the interaction of NSA5 with an ER transmembrane protein, SHISA5 (26). Human immunodeficiency virus 1 (HIV-1) replication can also be perturbed by autophagic degradation. Histone deacetylase 6 (HDAC6), in complex with the HIV restriction factor APOBEC3G, is responsible for the degradation of the virion infectivity factor Vif by autophagy (27). In addition to eliminate Vif factor, autophagy also selectively degrades both the transactivator Tat, involved in viral transcription, and the restriction factor tripartite motif-containing protein 5α (TIRM5α), respectively, in HIV-infected CD4^+^ T cells and Langerhans cells (28, 29).

Autophagy can also influence the immune antiviral responses. In infected plasmacytoïd dendritic (pDC) cells, autophagy favors the transport of viral genome of vesicular stomatitis virus (VSV) from the cytosol to TLR7-containing endosome, enhancing the type I interferon (IFN) production (15). Similarly, in mouse models of murine norovirus (MNV) infection, production of IFNγ-mediated antiviral responses require the autophagy elongation complex Atg12/Atg5/Atg16L1 (30). Importantly, the antiviral adaptive immune response can also be modulated by autophagy, mainly by promoting viral antigen presentation on class II major histocompatibility complex (MHC). That mechanism has been notably observed for Epstein-Barr virus (EBV), SINV, or HSV (4, 31).

While autophagy can restrict viral infection, some persisting viruses have developed several strategies to escape from the autophagic degradation, or even manipulate autophagy for their own benefit. The inhibition of autophagy induction has been noticed for a broad range of viral infections mainly by targeting Beclin-1. Indeed, HSV-encoded neurovirulence factor ICP34.5, viral homologues of Bcl-2, ORF16, or M11—respectively expressed by Kaposi’s sarcoma associated herpesvirus (KHSV) and murine γ-herpers virus (MHV) 68—and human cytomegalovirus (HCMV)-encoded TRS1 and IRS1 are all viral proteins capable to bind Beclin-1 and inhibit autophagy (32–35). mTOR pathway, an important negative regulator of autophagy initiation complex, can also be targeted by viruses to inhibit autophagy. A good example is the KSHV G protein-coupled receptor (vGPCR), which stimulates mTOR pathway and therefore inhibits autophagy in cells during KHSV infection (36).

In addition to target the initiation step of autophagy, some viral proteins also prevent the fusion of autophagosomes with lysosomes. HSV-1 encodes the TANK-binding kinase (TBK1) protein to prevent autophagic degradation, by regulating the autophagosome maturation through the phosphorylation of the autophagy receptors p62 and optineurin (37). Similarly, the K7 protein of KHSV promotes rubicon/beclin-1 interaction and inhibits the activity of PI3KC3, which leads to the blocking of the fusion of autophagosomes with lysosomes (38). In most of the cases, viruses with the ability to block autophagy maturation step can use autophagic vacuoles as support membranes for their own replication. The first example of virus that has been described to manipulate autophagy to enhance its replication is the poliovirus, for which autophagy inhibition decreases infection efficiency, whereas the autophagy inducer rapamycin increases it (39). Other picornaviruses, such as coxsackievirus B3 (CVB3) and foot-and-month disease virus (FMDV), also use autophagy to replicate (40, 41). HCV induces the formation of autophagosomes but blocks its fusion with the lysosome, which favors viral replication and virion production (42). Like K7 protein of KHSV, HCV-encoded NS4B induces rubicon expression, leading to the inhibition of autophagy maturation (43). Influenza virus (IAV) and human parainfluenza virus type 3 (HPIV3) can also trigger the accumulation of autophagosomes for viral replication, respectively, *via* the interaction of the viral protein M2 or M with LC3 (44, 45).

Indeed, manipulation of autophagy by many viruses confers double advantages for viruses: (i) it permits to avoid their degradation by inhibiting the autophagy influx, and (ii) it supplies to the viruses a double-membrane vacuole that could serve as a support for their replication.

## Oncogenic Viruses Modulate Autophagy to Favor Tumorigenesis

### Oncogenic Viruses and Tumorigenesis

Cancer is a multifactorial disease, combining genetic predispositions and environmental factors. Among all the elements promoting the cancer development, viruses can be directly linked with the formation of tumor. It is now well admitted that 15% of human cancers are caused by viruses (46). So far, only seven viruses have been demonstrated to have an oncogenic activity: Epstein-Barr virus (EBV), hepatitis B and C viruses (HBV and HCV), human papilloma virus (HPV), human T lymphotrophic virus type 1 (HTLV-1), Kaposi sarcoma-associated herpes virus (KSHV), and Merkel cell polyomavirus (MCPyV). Despite being very different in terms of tropism or viral structure and cycle, they share one characteristic: they are all responsible for persistent infections, criteria that seem to be essential to lead to virus-mediated oncogenesis. In order to persist and proliferate, viruses have to maintain their genome inside infected cell, prevent apoptosis of host cell while favoring its replication and escaping from the recognition of antiviral immune cells (47). To do that, oncogenic viruses encode proteins, called oncoproteins, that target and inactivate tumor suppressor proteins, such as retinoblastoma protein (pRb) or tumor protein 53 (p53) (48–52). p53 is a key transcription factor activated under stress responses, and it is responsible for cell cycle arrest or apoptosis induction. Similarly, pRb blocks cell cycle progression in G1 phase under stress conditions. HBV-encoded hepatitis B virus X (HBx), HTLV-1-encoded latency-associated nuclear antigen (LANA), and MCPyV-encoded large T antigen are all viral proteins that target and inhibit both p53 and pRb (52–55). Oncogenic viruses can also express proteins that target specifically p53 [such as E6 protein of HPV, latent membrane protein 1 (LMP1) of EBV, or NS5A protein of HCV] or pRb [including core protein of HCV, Epstein Barr virus latent 3C (EBNA3C) of EBV or E7 protein of HPV] (56, 57). Dysregulation of several cell cycle components, such as cyclins and cyclin dependent kinases (CDK), are also observed following oncogenic virus infection to bypass cell cycle checkpoints and pursue an infinite proliferation of infected cell. For example, E6 and E7 proteins of HPV have inhibitory activities on the CDK inhibitors p21 or p27 (58–60). Direct integration of viral genomic materials into the host genome may also modify expression of tumorigenesis-leading genes (61). Other studies demonstrated that oncogenic viruses have also developed strategy to escape from antiviral immune responses, inhibiting type I IFN response or cytotoxic activities of CD8^+^ T cells and natural killer (NK) cells (62–75).

### Oncogenic Viruses and Autophagy Inhibition

In precancerous cells, autophagy limits the formation of ROS, damages of DNA, and inflammation with the consequence of limiting tumorigenesis. This observation has been established in a lot of studies using different cancer models, where a decrease of autophagy activity has been observed in precancerous cells compared with non-tumor cells. Depletion of key autophagy genes has been associated with an increase of tumorigenesis in several tissues, leading to classify some autophagy genes as tumor suppressor genes. The first autophagy gene described to be involved in tumor formation is Beclin-1, for which a monoallelic depletion has been observed in various cancers (e.g., breast, ovarian, prostate, hepatocarcinoma, or lymphoma). Depletion of UVRAG in breast, colon, gastric, and prostate cancers also increases tumorigenesis (76–79). Similarly, knockout of both conjugated systems (Atg5/Atg7 or Atg3) favors tumorigenesis in liver tumor murine model, due to the accumulation of ROS in cells (80).

An increased range of studies highlighted that oncogenic viruses are able to modulate autophagy to persist in infected cells and/or favor the virus-induced tumorigenesis [extensively reviewed elsewhere (81–83)]. For instance, HPV inhibits autophagy during several steps of viral cycle. Cellular entry of type 16 HPV inhibits autophagy through the activation of autophagy-suppressor PI3K/akt/mTOR pathway, induced by the binding of viral proteins L1 and L2 to heparan sulfate proteoglycans (HSPGs) expressed on cell membrane (84). HPV-encoded E5 protein, by inhibiting p53, alters the transcription level of several autophagy genes involved in the formation of autophagosome (Beclin 1, atg5, atg7…), whereas E6 and E7 viral proteins inhibit the autophagy maturation step, observable by an accumulation of p62 (85, 86). In HPV-infected precancerous tissues and primary human keratinocytes, Mattoscio *et al.* have observed an accumulation of the autophagy receptor p62 in the cytosol of infected cells, suggesting an inhibition of autophagy in those cells (86). Similarly, in patients with oropharyngeal and oral cavity squamous cell carcinoma (SSC), HPV-positive tumors exhibit a lower level of LC3B as compared with HPV negative tumors, demonstrating that infection represses autophagy (87). Importantly, HPV-encoded E6 and E7 proteins inhibit autophagy maturation in anal tumor mice model, and this inhibition is associated with a greater susceptibility to HPV-induced anal carcinogenesis (88). Altogether, those studies strongly suggest that inhibition of autophagy during HPV infection actively participate in normal cells transformation.

HBV and HCV, the causative agents of hepatocellular carcinoma (HCC), have developed mechanisms to inhibit autophagy maturation, leading to the accumulation of autophagosomes inside infected cells and favoring the replication of viruses and the tumorigenesis (89–91). HBV-encoded oncogenic protein HBx prevents autophagosome maturation possibly by repressing the v-ATPase activity, and therefore by impairing the fusion of autophagosome with lysosome (92). This study highlights that HBx-mediated disruption of autophagic degradation may be critical for the development of HBV-associated HCC. Importantly, downregulation of autophagy in HBV-associated HCC patient specimens is inversely correlated with the expression of the microRNA-224 (miR-224), factor that promotes liver tumorigenesis. Moreover, the same study shows that miR-224 is preferentially recruited and degraded by autophagy, suggesting that HBx-mediated autophagy inhibition could promote HBV-associated tumorigenesis through the accumulation of miR-224 (93). While HBx protein has been shown to perturb the autophagic influx, another study observes an induction of HBx-mediated autophagy in HBV infected cells, which promotes the degradation of tumor necrosis factor receptor superfamily member 10B (TNFS10, called also TRAIL) and prevents the TNFS10-mediated antiviral immunity (94). Moreover, HBx-mediated autophagy has also been demonstrated to play an important role in NFκB-dependent production of pro-inflammatory cytokines, such as interleukin (IL)-6 or IL-8 that are two predictors of HCC progression in patients suffering from chronic hepatitis infection (95, 96). As previously described, HCV-encoded NS4B protein induces rubicon expression, leading to the inhibition of autophagosomes maturation (97, 98). Autophagy inhibition leads to an accumulation of the autophagy receptor p62 in HCV-associated HCC patients. Recent report also observed that an elevated expression of clusterin (CLU) in tumor tissues of HCV-HCC patients increases autophagy genes and upregulated p62 (99). Importantly, upregulation and phosphorylation of p62 activate the transcription factor Nrf2, involved in the metabolic reprogramming that promotes malignancy of HCV-positive HCC (100).

Viral cycles of KSHV and EBV, which belong to the herpesvirus family, are divided into two phases : a latency and a lytic phase (101, 102). Oncogenic properties of these viruses are linked with the latency phase, with a cell proliferation promoted and a cell survival increased. Interestingly, it has been shown that during this phase, autophagy is mainly inhibited. For KSHV infection, autophagy inhibition is led by both the viral FLICE-like proteins FLIP (vFLIP) through the repression of the conjugated system LC3 and LANA viral protein by preventing the downstream autophagy genes expression related to p53 (52, 103, 104). Autophagy is also inhibited during the lytic phase of KHSV infection. Expression of viral G-protein-coupled receptors (vGPCR) promotes the autophagy repressor pathway PI3K/AKT/mTOR as well as the degradation of the autophagic protein ATG14L, and viral bcl2 (vBcl2) represses Beclin-1 activation (105–107). Moreover, as previously mentioned, K7 protein of KSHV leads to the inhibition of autophagy maturation (108). The autophagy-associated protein p62 can also interfere with the lytic phase of KSHV, as an inhibition of exportin 1 (XPO1) induced retention of p62, which enhanced expression of innate immune-related genes (e.g., IRF7, ISG15, IFIT1, IFIT2, and IFIT3) that leads to a reduction of KSHV lytic replication (109). While further investigations are required to decipher the relation linking autophagy inhibition and oncogenesis in KSHV infected cells, one study showed that KSHV-mediated subversion of autophagy perturbs senescence and facilitates the proliferation of infected cells, two processes involved in cell tumorigenesis (110). Similarly, in EBV-infected cell, latency protein LMP-2 promotes mTOR pathway and inhibits autophagy (111). Recent study also demonstrates that EBV infection blocks the autophagic flux to favor its replication through the activation of two viral Bcl-2 homologue proteins, BHRF1 and BALF1 (112, 113). This impairment of complete autophagy leads to an accumulation of p62 in the nucleus of EBV-transformed lymphoblastic cell lines (LCLs) that promotes oxidative stress and limits the efficiency of DNA damage response, processes that could enhance virus-mediated oncogenesis (114). Recent investigation also suggested that autophagy induction, mediated by the upregulation of CXCR4 in EBV-associated gastric carcinoma (EBVaGC), protects tumor cells from apoptosis and promotes replication of EBV (115). Interestingly, publications converge to show that modulation of autophagy in EBV-infected tumor cells supports the replication of viruses and favors persistent latent infection, two processes that could facilitate tumorigenesis.

HTLV-1 also encodes an oncoprotein, called Tax-1, that modulates autophagy by acting both on autophagy initiation and maturation steps (116). In HTLV-1 transformed T or in Tax-immortalized CD4 memory T cells, Tax-1 facilitates autophagic initiation by activating IκB kinase complex, which subsequently recruits and activates the autophagy initiation complex Beclin-1/PI3KC3 (117). Tax-1 also blocks the fusion of autophagosomes with lysosomes, leading to an accumulation of autophagic vacuoles in the cytosol that promotes HTLV-1 production (118). It is interesting to note that another study has observed that HTLV-1-encoded basic leucine-zipper factor (HBZ) promotes the activity of mTOR pathway, which could be an additional autophagic suppressor mechanism in infected cells (119). These results indicate a critical role of HTLV-1-deregulated autophagy in promoting survival and transformation of T cells infected by the virus.

MCPyV, the causative agent of Merkel cell carcinoma (MCC), reduces autophagy level in infected cells dependently of the viral oncogenic large antigen (LT-ag) protein. LT-ag favors the expression of miR-30a-3p, miR-30a-5p, and miR-375, which target and repress the level of expression of key autophagy genes (*Beclin-1*, *atg7*, and *p62*) (120). In MCC tumor, low expression of ATG7 and p62 are correlated with MCPyV-positive tumor, suggesting the importance of autophagy evasion in MCPyV-associated tumorigenesis. Moreover, Torin-1 treatment (an mTORC1 inhibitor) induces cell death of MCC, which can be reduced by autophagy inhibitors, suggesting that MCPyV oncoproteins suppress autophagy to protect cancer cells from cell death (120).

## Oncolytic Viruses Enhance Autophagy to Increase Tumor Cell Death and Immunogenicity

Oncolytic viruses (OVs) have recently emerged as a promising cancer therapeutic approach with great potential for the treatment of a broad spectrum of cancer, especially tumors that have acquired drug resistance to the first-line chemotherapeutics. OVs have been selected or designed to specifically target and kill cancer cells, with low replication capacity in normal tissues (121, 122). The mechanisms of tumor selectivity are multiple, including the expression of viral genome through the cancer-specific gene overexpression involved in the transcriptional element, the specific expression of virus receptor by the cancer cells, the overactivity of metabolic capacity in tumor cells (which is required for virus replication) but also tumor-specific defects of antiviral immunity [extensively reviewed elsewhere (123)]. Several interesting OVs are under investigation in preclinical and clinical studies, including coxsackievirus, adenoviruses (AdV), herpes simplex virus (HSV), Marada virus, Measles virus (MeV), Newcastle disease virus (NDV), vesicular stomatitis virus (VSV), parvovirus, and vaccinia virus (123). Direct lysis of tumor cells along with indirect induction of antitumoral immunity against specific tumor antigens are the two ways by which OVs limit tumor progression.

### Oncolytic Viruses and Autophagy-Dependent Cell Death

Paradoxically, although autophagy is well recognized as a cell survival process that promotes tumor development, it can also participate in a caspase-independent form of programmed cell death. Due to the important role of autophagy in cell survival and the close relationship between autophagy and other types of cell death (e.g., apoptosis or necrosis), classification of autophagy as type of programmed cell death has been largely discussed and debated during the last decade [extensively reviewed elsewhere (124)]. However, it is now admitted that autophagy-dependent cell death (ADCD) is a type of cell death that requires autophagy and/or autophagy components and for which the inhibition of autophagy machinery and/or components, genetically or chemically, prevents cell death (125). ADCD has been firstly described to mediate physiological cell death *in vivo*, during the developmental program of *D. melanogaster* (126), but autophagy also appears to be involved in the death of many cancer cells in response to several therapies, especially in tumors with the lack of crucial apoptotic modulators (e.g., BAX and BAK or caspases) [extensively reviewed elsewhere (127)].

**Figure 3:**
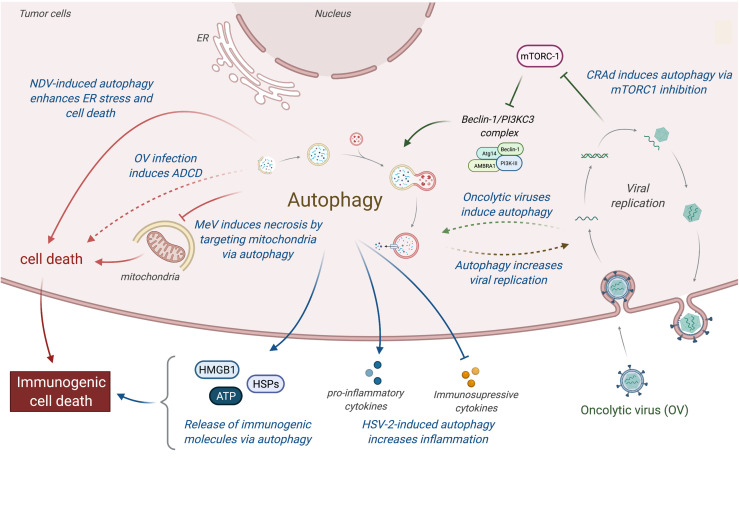
Relations between oncolytic viruses and autophagy in tumor. Oncolytic viruses induce an immunogenic cell death by inducing autophagy, which favors the cell death of tumor-infected cells and the release of immunogenic molecules (e.g., HMGB1, ATP, or HSPs proteins). More specifically, CARds replication induces autophagy by an inhibition of mTORC1, which enhances the induction of cell death and the release of immunogenic molecules. MeV induces mitophagy to eliminate mitochondria, resulting to a decrease of apoptosis and an increase of necrosis, a more immunogenic cell death. NDV infection increases ER stress, resulting to an induction of autophagic cell death and the secretion of immunogenic molecules. In addition, HSV-2 infection can also perturb the tumor microenvironment by favoring the expression of pro-inflammatory molecules (e.g., TNFα, IL-1β; and GM-CSF) and limiting the production of immunosuppressive molecules (e.g., IL-10 and TGFβ). CRads, Conditionally Replicating Adenoviruses; MeV, Measles Virus; NDV, Newcastle Disease Virus; HSV-2, Herpes Simplex Virus 2; ADCD, autophagy-dependent cell death.

Oncolytic viruses destroy tumor cells by inducing different types of cell death. For example, parvovirus and NDV exert oncolytic activities by triggering apoptotic pathways in many cancers, while vaccinia virus leads to programmed necrotic cell death in ovarian and colon infected tumor cells (128–131). In addition, some oncolytic viruses are also able to induce tumor cell lysis through autophagy induction (**Figure 3**) (132).

A number of different conditionally replicating adenoviruses (CRAds) have been shown to induce autophagy in tumor cells (133). In nude mice subcutaneously transfected with glioma tumor cells, adenoviruses expressing the adenovirus early (E)1A gene (which leads to viral replication) under the control of the human telomerase reverse transcriptase promoter (hTERT-Ad) kill telomerase-positive tumor cells by inducing autophagic cell death, notably by inhibiting the mTORC1 activity in infected cells (134). A more recent study showed that OBP-31, an oncolytic adenovirus that is derived from hTERT-Ad, induces the autophagic cell death of human glioma tumor cells through an E2F1-mir7-EGRF pathway (135). Similarly, CRAds, which use the survivin promoter, enhance autophagy in glioma infected cells and favor the elimination of tumor cells *via* a beclin-1 dependent mechanism (136). Delta-24-RGD, an oncolytic adenovirus that selectively replicates in cancer cells with an abnormal Rb pathway, induces an autophagy-dependent cell death of brain tumor stem cells and improves the survival of glioma-bearing mice (137). *In vitro* experiments also showed that wild-type or E1-deficient adenoviruses induce autophagy in lung, cervical, and colon cancer cells and that autophagy induction correlates with an increase of viral replication and tumor cell death (138).

The oncolytic HSV-1 strain RH2, which lacks the *γ34.5* gene, induces the cell death of squamous cell carcinoma (SSC). While no significant changes were observed using caspase inhibitors, the cytotoxicity of RH2 infection was inhibited when infected tumors cells were treated with autophagy inhibitors (3-methyladenine and bafilomycin), demonstrating that HSV-1/RH2 induces autophagic cell death in SCC cells (139). In gastric carcinoma, an excessive endoplasmic reticulum stress was observed in Newcastle disease virus (NVD)-infected cancer cells, triggering autophagy and cell death (140). Similarly, Meng et al. (2012) reported that infection of malignant U251 cells with NDV boosts the formation of autophagosomes, which facilitates the replication of the virus (141). Interestingly, pharmacological modulation of autophagy was investigated in order to enhance the oncolytic potential of NDV strain FMW (NDV/FMW) in drug-resistant lung tumor cells (A549 resistant to cisplatin or paclitaxel). Combination of NDV with chloroquine or rapamycin significantly promoted the oncolytic efficiency of NDV/FMW in lung cancer bearing mice (142). Edmonton strain of MeV (MeV-Edm) exploits selective autophagy to increase its replication in non-small-cell lung cancer (NSCLC) by targeting the degradation of mitochondria *via* autophagy (mitophagy), which results in a decrease of innate immune response by limiting the production of RIG-I like receptor (RLR)-dependent type-I interferon production (143). Another study showed that the persistent viral replication mediated by MeV-Edm-induced mitophagy prevents apoptosis but at the end leads to necrotic cell death (144), suggesting that MeV-induced autophagy could switch from apoptosis to a more immunogenic cell death in NSCLCs following the infection.

An attractive strategy among virus-based oncolytic system is to design viral vectors that express pro-autophagic genes, in which gene-virotherapy approach significantly enhances tumor cell death by activating autophagy, especially in tumors that have acquired resistance for apoptosis. Several studies have investigated the therapeutic effect of engineered recombinant OV expressing *beclin-1* gene. For example, the oncolytic adenovirus expressing *beclin-1* (SG511-BECN) infection induces a significant autophagic cell death in a variety of leukemic cell lines and primary leukemic blast (145). Interestingly, SG511-BECN induces cell death of both acute and chronic myeloid leukemia, but has less cytotoxicity in normal cells. In murine leukemia model, SG511-BECN prolongs mice survival and decreases the xenograft tumor size by inducing autophagic cell death (145). Recently, oncolytic vaccinia virus that expresses Beclin-1 (OVV-BECN) was also tested for its *in vitro* and *in vivo* oncolytic activity in blood cancer (146). OVV-BECN induces a significant autophagic cell death in both wild-type leukemia and multiple myeloma cells lines and has a greater antitumor activity compared with the wild-type vaccinia virus, demonstrating a favorable therapeutic effect of autophagy in vaccinia-based treatment of blood cancers.

### Oncolytic Viruses and Autophagy-Mediated Immunogenic Cell Death

Besides their direct killing potential, oncolytic viruses require the activation of immune responses to be long-lasting effective against cancer. Most of the OVs, including HSV-1, MeV, adenoviruses, or NDV, have been described to induce an immunogenic cell death (ICD), which is critical for their virotherapy efficacy (147). ICD is a type of cell death that is sufficient to induce an adaptive immune response against exogenous or endogenous antigens expressed by dying cells and elicited by the presence of danger associated molecular patterns (DAMP). While pathways of ICD induction could be multiple, it is often related to endoplasmic reticulum (ER) stress and reactive oxygen species (ROS) production (148). The main hallmarks of ICD that have been described so far include ecto-expression of calreticulin (which is normally expressed in ER), ATP release, high-mobility group box (HMGB) 1 release in different redox states but also the activation of annexin 1, the secretion of type I IFN and IL-1β, or the exposure of F-actin and heat shock protein (HSP-70 and HSP90) (147). ICD leads to the activation of adaptive immune cells, including dendritic cells (DC) and T lymphocytes (LT). OVs are one of the most described ICD inducers, and infected tumor dying cells are often associated with the presence of danger signals (ATP and HMGB1 release or CRT exposure), the secretion of pro-inflammatory cytokines, as well as the release of tumor associated antigens (TAA), which lead to a strong activation of antitumor immune responses (149–153).

Pathways of ICD induction vary a lot between viruses, but mounting evidences indicate that autophagy plays a critical role in that induction. One of the first examples highlighting the importance of autophagy in ICD has been observed during embryonic development. During embryonic cavitation, embryonic bodies (EBs) derived from cells deficient for autophagy genes (*atg5* or *beclin-1*) fail to cavitate. This defect is due to the persistence of cells corps during the embryonic development, which is the result of the absence of “eat-me” signal (exposure of phosphatidylserine) and the poor release of ATP in autophagy-deficient EBs (154). In tumor model, Michaud et al. reported that autophagy is required for the immunogenicity of chemotherapy-induced cell death, as autophagy-deficient dying cells fail to release ATP and subsequently to attract DC and LT into the tumor bed in colon carcinoma mice model (155). Similarly, it has been observed that radiotherapy of lung and colon cancer elicited an anticancer immune response that was dependent on autophagy-induced ATP release from dying cells, release that is required for a dense recruitment of lymphocytes into the tumor site (156). In epithelial and glioblastoma cancer cells, autophagy regulates the passive HMGB1 release from dying cells and active HMGB1 secretion (157, 158). Strikingly, the close correlation between autophagy induction and ICD properties observed during antitumor cytotoxic agents treatment in blood cancers supports the relationship between autophagy and ICD (158). In addition to favoring the exposition of DAMP from dying cells, autophagy can also promote antigen presentation from cancer cells to DCs and subsequently to T cells, a necessary step for the implementation of a robust antitumor immune response. Indeed, autophagy promotes the presentation of antigens not only by major histocompatibility complex (MHC) class II but also by MHC class I, as observed for endogenous viral antigens during HSV-1 infection or during influenza infection of tumor cells, and for cross-presentation of TAA from uninfected tumor cells (159, 160).

Oncolytic adenoviruses have been shown to induce an autophagy-dependent ICD, leading to the release of a great number of DAMP molecules and TAAs (137). In prostate cancer cells, the combination of Ad5/3 fiber-modified oncolytic adenovirus armed with granulocyte macrophage colony-stimulating factor (Ad5/3-D24-GMCSF) with low-dose of temozolomide (a chemotherapeutic agent) results in the increase of intracellular level of autophagy in tumor dying cells and favors immunogenic cell death, as indicated by elevated calreticulin exposure, ATP secretion, and HMGB1 release (161). A more recent report demonstrated that oncolytic adenovirus-induced autophagy is critical for the processing and the presentation of TAAs incorporated into viral capsid protein on MHC class II, suggesting that the combination of adenoviruses with autophagy inducers may enhance the antitumor immune responses (162). NDV infection also induces a strong ICD in lung and glioblastoma cancer cells, observable by the elevated exposure of calreticulin, the release of HMGB1, ATP, and HSP79/90, and the induction of a long-term tumor-specific immune response (163, 164). A recent study has shed light on the important role of autophagy in NDV-mediated ICD in lung cancer, as the depletion of autophagy-related genes, in contrast to inhibition of apoptosis or necrosis, significantly inhibits the induction of ICD determinants by NDV infection (165). In addition to increase the DAMP or TAAs expression by tumor dying cells, autophagy can also modify the tumor microenvironment. Using the herpes simplex virus type 2 (HSV-2)-based oncolytic virus ΔPK, one report has demonstrated that autophagy promotes the release of proinflammatory cytokines (TNFα, IL-1β; and GM-CSF) through the TLR2 activation, and that contributes to the inhibition of tumor immunosuppressive microenvironment in melanoma cells (166).

## Other Correlations Between Viral Infection, Autophagy, and Tumor Progression

In addition to favoring the tumorigenesis and the immunogenic cell death, modulation of autophagy by viral infections could also influence other steps of tumor development, including tumor cell survival, proliferation, migration, and resistance to antitumor therapies, as well as immune response efficiency.

### Tumor Cell Survival

When the tumor is established, the impact of autophagy on tumor growth and/or clinical features of patients remains unclear and seems to be greatly influenced by the type and/or the stage of the disease. However, several studies described that autophagy has pro-tumor effects, promoting the tumor cell survival, the proliferation, the migration, and the resistance to radio- and chemotherapies (**Figure 2**) (167). One of the most obvious involvement of autophagy in tumor growth is the fact that autophagy could recycle non-essential cytoplasmic elements to support tumor cell survival. Indeed, the more the tumor grows, the more the cells are moved away from the vascular system, leading cells under hypoxic and/or starvation condition (168). Both starvation and hypoxia have been demonstrated to induce a strong autophagy response in tumor cells, and this autophagy acts as recycling mechanism to promote stress tolerance and to supply metabolic precursors for the tumor cell survival (169, 169). Interestingly, some common oncogenic gene mutations (e.g., *ras*) have been shown to increase autophagy in order to maintain tumor cell survival (170).

**Figure 4:**
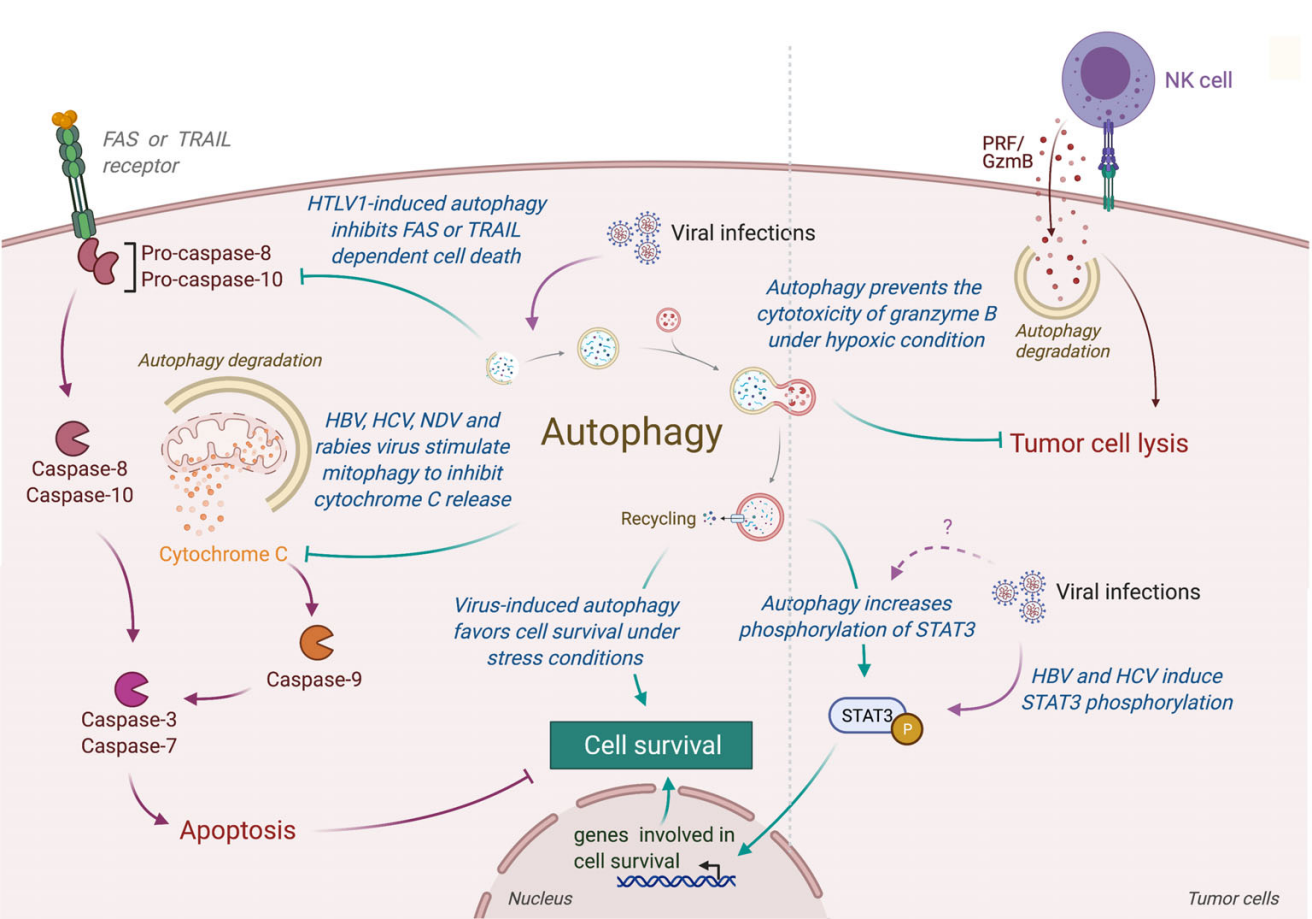
Role of virus-induced autophagy in tumor cell survival. Viral infections can induce autophagy in tumor cells, leading to a protection of tumor cells from stress-induced or immune cells-induced cell death. In one hand, autophagy protects tumor infected cells from stress conditions (e.g., starvation or hypoxia) by limiting the accumulation of damaged organelles or by increasing the expression of genes involved in cell survival (e.g., *via* the phosphorylation of SATA3). In another hand, virus-induced autophagy limits the induction of apoptosis, protecting tumor cells from death receptor- or mitochondria-mediated cell death. Autophagy also takes part in the resistance of infected tumor cells from immune cell lysis by targeting and neutralizing granzyme B activity. HTLV-1, Human T cell Leukemia/lymphoma Virus type 1; HBV, Hepatitis B Virus; HCV, Hepatitis C Virus; NDV, Newcastle Disease Virus; PRF/GzmB, Perforin/Granzyme B.

Autophagy could also limit the induction of programmed cell death, including apoptosis, by the selective removal of damaged organelles (e.g., damaged mitochondria) or by the selective elimination of the pro-apoptotic signal transduction [extensively reviewed elsewhere (171)]. Interestingly, several viruses have been observed to promote survival of infected cells by repressing apoptosis through an autophagy-dependent mechanism (**Figure 4**) (172–179). In human and mouse neuroblastoma cells, wild-type rabies virus limits apoptosis in infected cells by inducing a complete autophagic influx (179). The overexpression of HBV protein HBx and HCV protein NS5A can both reduce the starvation-induced cell death through the activation of autophagy and the inhibition of mitochondrial apoptosis, leading to the tumor cell survival of respectively advanced HCC and hepatoblastoma cells (174, 175). Similarly, the Tax protein of human T-cell leukemia virus type 1 (HTLV-1) favors the resistance of astroglioma infected cells for FASL-mediated and TRAIL-mediated apoptosis by increasing autophagy (176). In lung tumor, NDV-induced autophagy promotes viral replication and tumor cell survival by preventing cancer cells from caspases-dependent apoptosis, recruiting p62-mediated autophagy to control cytochrome c release from mitochondria (180).

Contribution of autophagy in tumor survival has also been illustrated by the fact that autophagy could prevent lysis of tumor cells from antitumor cytotoxic immune cells. For instance, autophagy has been reported to play an important role in hypoxia-induced resistance of lung tumor cells to cytolytic T lymphocyte-mediated lysis (181). Indeed, inhibition of Atg5 or Beclin-1 reduce the phosphorylation of STAT3, an important transcription factor involved in tumor survival and resistance to T cell cytotoxicity. Similarly, Viry *et al.* revealed that hypoxia-induced autophagy is responsible for the degradation of granzyme B in tumor cells delivered by NK cells, leading to their resistance to NK-mediated cell death (182). Interestingly, similar STAT3 modulation has been reported in infected cells (183). Given the fact that these proteins are also responsible for autophagy-mediated resistance to starvation in tumor, it will be very interesting to investigate the impact of virus-induced autophagy on the resistance to antitumor immunity.

### Metastasis Formation

Autophagy has also been associated with metastasis formation. Actually, the role of autophagy in metastasis occurrence is controversial (184). In one hand, autophagy has been shown to limit metastasis of some tumor cells. For instance, autophagy decreases the glioblastoma tumor cells migration and invasion, reversing the epithelial-mesenchymal transition (EMT) (185). Similarly, autophagy has been associated with the selective degradation of MET/HGF receptor tyrosine kinase, two kinases involved in cell invasion, therefore inhibiting cancer cell lines mobility (186). In the other hand, autophagy has been described to be essential for the resistance to cell-detachment-induced apoptosis, called anoikis (187). For example, the group of Jian Fan demonstrated that the inhibition of autophagy suppresses pulmonary metastasis of hepatocellular carcinoma in mice through impairing anoikis resistance (188). Emerging evidence show that autophagy not only enhances the survival of disseminating tumor cells but also promotes the survival and the maintenance of a stem-like subpopulation of tumor cells that drives invasion, treatment resistance, and cancer recurrence (189). In addition, several autophagy substrates have been shown to be involved in the regulation of the epithelial-mesenchymal transition (EMT), tumor cell migration, and invasion [extensively reviewed elsewhere (190)].

Viral infections and virus-related molecules are also known to promote metastasis formation. EBV has been shown to encode several mature miRNAs, where some of them have been demonstrated to promote tumor development by targeting virus-infected host genes or self-viral genes. Several studies demonstrated that these miRNA could promote EMT, migration and metastasis of the nasopharyngeal carcinoma (NPC) cells (191–195). HPV16 E6 protein expression has been shown to increase actin polymerization through the degradation of Na+/H+ exchanger regulatory factor 1 (NHERF1) protein, facilitating the migration of the cervical cancer cell and the development of metastasis (196). Viral proteins E7 of HPV16 and E1 of HCV have been shown to inactivate the tumor metastasis repressor Nm23-H1 in human keratinocyte HaCaT cell line and HCC cells line, respectively, increasing metastasis formation (197, 198). HSV2, HBV, or HCMV related-proteins or miRNA have also been demonstrated to promote metastasis development (199–201). Interestingly, HCMV-mediated promigratory signal requires the activation of Ras homolog family member A (RhoA), a protein involved in cell migration that can be modulated by autophagy (202). A recent report showed that under HBV infection, HBx-induced autophagy promotes the expression of long non-coding RNA activated by transforming growth factor (TGF)−β (lncRNA−ATB) and TGF−β, two key actors for the migration and invasion capabilities of liver cells (203).

While the role of autophagy in virus-induced metastasis needs to be further analyzed to understand the actual function of this mechanism in tumor development, it is interesting to notice the increasing number of evidences that report a strong correlation between viruses capable to modulate autophagy and their capacity to increase tumor cell migration and metastasis development.

### Resistance to Antitumor Therapies

Another aspect by which autophagy favors the tumor development concern its impact on the resistance to antitumor treatments. In various tumor models (e.g., ovarian, colon, or osteosarcoma tumors), autophagy has been demonstrated to be an important actor of chemoresistance, leading to an increase of the survival of tumor cells under treatment of salt-based chemotherapies (204–208). Autophagy has also been observed to be an important promoter of resistance to radiotherapy in various tumor models, including lung, glioma, pancreatic, and colorectal cancer (209–212). Interestingly, in order to overcome this resistance, several therapeutic approaches consisting in combining chemotherapy and radiotherapy with autophagy inhibitors have emerged with very promising results (213, 214).

Viruses or viral proteins have also been linked with the resistance to different antitumor therapies (215–220). While mechanisms involved in viruses-mediated resistance to therapies differ with the type of viral infection, the main strategy consists in inhibiting apoptotic cell death in infected tumor cells. EBV miR BART20-5p has been observed to favor chemoresistance to 5-FU and docetaxel in gastric cancer by targeting the pro-apoptotic BCL2-associated agonist of cell death (BAD) expression (221). EBV, though the viral LMP1 protein, has been demonstrated to decrease the expression of the two pro-apoptotic factors PDCD4 and FasL, leading to a chemoresistance to cisplatin in nasopharyngeal carcinoma cells (222). EBV has also been shown to induce chemoresistance to 5-FU in gastric cancer cells by decreasing the cleavage of PARP and caspase 3 and increasing the anti-apoptotic Bcl2 expression (223). In nasopharyngeal carcinoma, EBV-encoded miR-BART4 and miR-BART8-3p favor resistance to radiotherapy by inhibiting apoptosis (224, 225). EBV latent viral protein LMP1 has also been demonstrated to induce radioresistance by preventing DNA Damage Response (DDR) through the phosphorylation of AMPK (thr^172^) and inhibiting therefore its interaction with DNA-dependent protein kinase (DNA-PK), required for the DDR (226). While autophagy has not been investigated in this study, it is interesting to note that AMPK activity is an important regulator of autophagy, and AMPK-induced autophagy could help to the EBV induced radioresistance. Similarly, a recent study demonstrated that LMP1 induces autophagy *via* the binding of BCL2/adenovirus E1B 19 kDa protein-interacting protein 3 (BNIP3) to beclin-1 (227). This autophagy stimulation has been shown to enhance the resistance of LMP1-positive nasopharyngeal carcinoma (NPC) cells against irradiation by protecting tumor cells from apoptosis. M. Antonioli et al. also demonstrated that HPV infection in oropharyngeal squamous cell carcinoma sensitize tumor cells to cisplatin-induced apoptosis by inhibiting autophagy, supporting the idea that modulation of autophagy during acute or latent infections could impact the resistance of tumor cells to antitumor therapies (228).

### Antitumor Immune Responses

**Figure 5:**
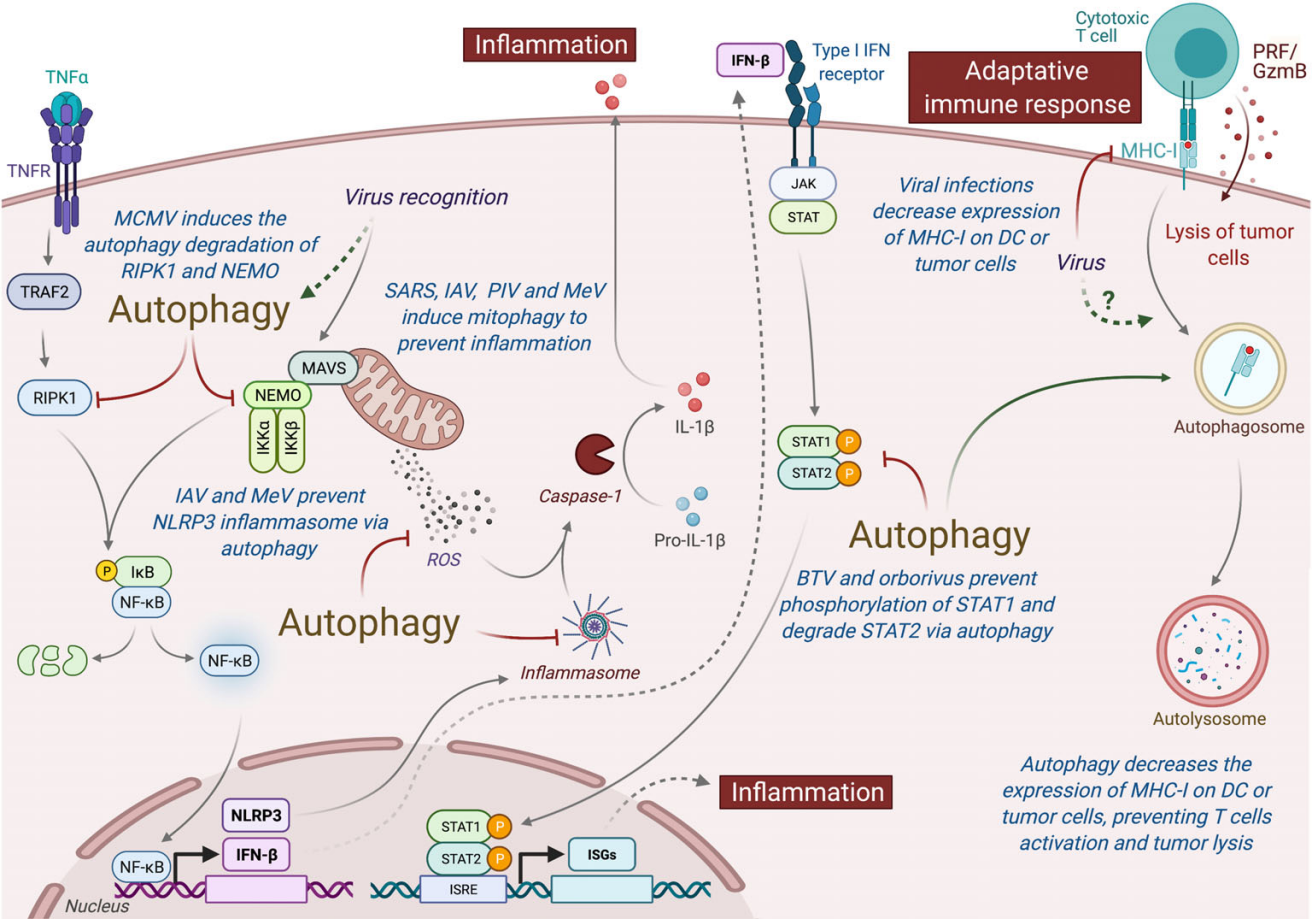
Autophagy induced under viral infection can perturb antitumor immunity. Autophagy can interfere with innate and adaptive immunity and impact the antitumor immune responses. Viruses-induced autophagy perturbs type I IFN pathway by limiting NF-κB activation and STAT1/STAT2 phosphorylation. Several viruses (e.g., SARS, IAV, PIV, and MeV) can also modulate mitochondria activity *via* mitophagy and prevent inflammation by inhibiting the release of ROS or by preventing the formation of inflammasome. Viral infections or autophagy can also modulate the expression MHC-I by tumor cells or DC, limiting the action of antitumor cytotoxic T cells. MCMC, Murine Cytomegalovirus; IAV, Influenza A Virus; MeV, Measles Virus; SARS, SARS-Coronavirus; PIV, Parainfluenza Virus; BTV, Bluetongue virus; MHC-I, Major Histocompatibility Complex I; PRF/GzmB, Perforin/Granzyme B, DC, dendritic cells.

Type I interferon (IFN-I) is an important class of pro-inflammatory cytokines produced in response to viral infection and other environmental stresses. While IFN-I is very important for efficient virus clearance, emerging studies have shown that these proteins are also a very important driver for antitumor immunity, promoting the efficiency of immune cells to eliminate tumor [extensively reviewed elsewhere (229)]. Many intracellular pathways can recognize viral components and lead to the production of IFN-I, including the stimulation of RIG-I like receptor (RLR) and mitochondria antiviral signaling protein (MAVS), present in the outer membrane of mitochondria. Autophagy, notably through the targeting of mitochondria (mitophagy), is able to regulate RLR-mediated IFN-I production (230). Several viruses induce mitophagy to impair the type-I interferon production, including the SARS-Coronavirus, the IAV and parainfluenza virus or MeV (**Figure 5**) (231–234). Damaged mitochondria can also release danger signals, including the reactive oxygen species (ROS) and mitochondrial DNA, which in return can activate inflammasome and the release of pro-inflammatory cytokines that could perturb tumor immunity (235, 236). Interestingly, some viruses, including IAV and MeV, have been also shown to induce mitophagy to prevent NLRP3-mediated inflammation (237, 238). Autophagy could also prevent virus-induced IFN-I production by other mechanisms. Induction of autophagy by HPV16 E7 protein is responsible for the autophagic degradation of STING protein, which impairs IFN-I production and induces important deregulation of tumor immunity and promotion of Head and Neck Squamous cell carcinoma (239). Similarly, Bluetongue virus (BTV), an orbovirus targeting ruminants, impairs IFN-I pathway by preventing STAT1 phosphorylation and by degrading STAT2 through autophagy (240). Inhibition of autophagy maturation in HCV-infected hepatocytes, through the expression of Rubicon, was recently associated with an increase of expression of type I IFN-related genes and an upregulation of HCV replication (241). Other pro-inflammatory cytokines, important for tumor immunity, could also be regulated by virus-induced autophagy. As a good example, MCMV M45 protein induces the autophagic degradation of two cellular signaling proteins involved in nuclear factor κ-light-chain-enhancer of activated B cells (NF-κB)-mediated cytokine production, the NF-κB essential modulator (NEMO), and the receptor-interacting protein kinase 1 (RIPK1) (242, 243).

In addition to limit pro-inflammatory cytokine production, viruses have elaborated strategies to escape from the cytotoxic immune cells. To avoid the recognition and elimination of infected cells by cytotoxic lymphocytes, many viruses decrease the major histocompatibility (MHC) class I antigen presentation pathway [extensively reviewed elsewhere (244)]. Similar strategy has also been observed in many cancer cells. The decrease of MHC-I expression has also been observed in dendritic cell (DC) infected with HIV, MCMV, or varicella zoster virus, and it has been associated with a reduction of their capacity to stimulate T cell proliferation (245). Interestingly, autophagy has been associated to a decrease of MHC-I expression on DC by facilitating the endocytosis and the degradation of MHC-I (246). A recent study demonstrated that autophagy regulates the expression of MHC-I in pancreatic tumor cells, as the inhibition of the autophagy machinery increased MHC-I expression on tumor cells, enhanced antitumor T cell response, and reduced tumor growth in mice model (247).

## Conclusion

Since several years, investigations on autophagy process shed light many biological pathways to an autophagy-dependent modulation. To understand the role of autophagy in host cells, it is important to consider the physiologic context, as autophagy could have distinct functions according to the biological model and/or the stage of the disease. During the initial step of tumor development, cells accumulate damages that disturb key molecules involved in cell cycle and promote tumorigenesis. Both tumor suppressor genes and oncogenes are implicated in autophagy regulation, linking autophagy directly to cancer occurrence. Recent discoveries showed that oncogenic viruses, which lead to the transformation of precancerous cells to malignant tumor, inhibit autophagy by targeting specifically autophagy molecules. Inhibiting autophagy in infected cells favors the accumulation of damaged organelles and/or proteins in host cells and therefore promotes the accumulation of stress components and genetic mutations.

In another hand, autophagy induced in more advanced tumor is more prone to help tumor growth. As a cellular degradative process, autophagy helps proliferative tumor cells for their nutrient supply and has an important prosurvival function, favoring tumor cell resistance to several therapies. Autophagy may also promote metastasis by protecting detached and stressed tumor cells as they travel through blood vessels and establish new colonies at distant sites. Importantly, many viruses capable to modulate autophagy have similar impact on tumor behavior, and while the direct link between autophagy and tumor development has not been well established in all studies, strong evidences have suggested an important connection between autophagy and virus-mediated tumor modifications. However, further investigations are needed to clarify autophagy’s modulation by viral infections and tumor development. Considering the role of autophagy in aberrant cell physiology, the understanding of these molecular processes is crucial for the development of new therapeutics against cancers and potentially other proliferative diseases.

## Author Contributions

LL and P-EJ performed similar bibliography. LL and P-EJ equally contributed to write the manuscript. P-EJ performed figures and supervised this work. IC, SS and MA helps authors during the conception and correction of the manuscript. All authors contributed to the article and approved the submitted version.

## Funding

This work was supported by the “Institut National de la Sante et de la Recherche Medicale” (INSERM), Sorbonne Université and Université de Paris.

## Conflict of Interest

The authors declare that the research was conducted in the absence of any commercial or financial relationships that could be construed as a potential conflict of interest.

## Publisher’s Note

All claims expressed in this article are solely those of the authors and do not necessarily represent those of their affiliated organizations, or those of the publisher, the editors and the reviewers. Any product that may be evaluated in this article, or claim that may be made by its manufacturer, is not guaranteed or endorsed by the publisher.
